# Glass/Au Composite Membranes with Gold Nanoparticles Synthesized inside Pores for Selective Ion Transport

**DOI:** 10.3390/ma13071767

**Published:** 2020-04-09

**Authors:** Denis Lebedev, Maxim Novomlinsky, Vladimir Kochemirovsky, Ilya Ryzhkov, Irina Anfimova, Maxim Panov, Tatyana Antropova

**Affiliations:** 1 Saint Petersburg State University, 13B Universitetskaya Emb., St Petersburg 199034, Russia; ooc41hmo@mail.ru (M.N.); v.kochemirovsky@spbu.ru (V.K.); m.s.panov@spbu.ru (M.P.); 2 Institute of Computational Modelling SB RAS, Akademgorodok 50/ 44, Krasnoyarsk 660036, Russia; rii@icm.krasn.ru; 3 Siberian Federal University, Svobodny 79, Krasnoyarsk 660041, Russia; 4 Grebenshchikov Institute of Silicate Chemistry (ISCh) RAS, 2 Adm. Makarova emb., St Petersburg 199155, Russia; anfimova-i@mail.ru (I.A.); antr2@yandex.ru (T.A.)

**Keywords:** porous glass, membrane, gold nanoparticles, laser synthesis, ion transport, modelling

## Abstract

Nanocomposite membranes have been actively developed in the last decade. The involvement of nanostructures can improve the permeability, selectivity, and anti-fouling properties of a membrane for improved filtration processes. In this work, we propose a novel type of ion-selective Glass/Au composite membrane based on porous glass (PG), which combines the advantages of porous media and promising selective properties. The latter are achieved by depositing gold nanoparticles into the membrane pores by the laser-induced liquid phase chemical deposition technique. Inside the pores, gold nanoparticles with an average diameter 25 nm were formed, which was confirmed by optical and microscopic studies. To study the transport and selective properties of the PG/Au composite membrane, the potentiometric method was applied. The uniform potential model was used to determine the surface charge from the experimental data. It was found that the formation of gold nanoparticles inside membrane pores leads to an increase in the surface charge from −2.75 mC/m^2^ to −5.42 mC/m^2^. The methods proposed in this work allow the creation of a whole family of composite materials based on porous glasses. In this case, conceptually, the synthesis of these materials will differ only in the selection of initial precursors.

## 1. Introduction

Due to development of modern technologies, the number of studies of ion transport in membranes has increased significantly in recent years. In turn, membrane technologies have found applications in important fields of science and industry, such as water treatment [1,2]; separation of mixtures and production of pure substances [3,4]; electrochemical energy conversion and storage devices [5,6]; chemical sensors and biosensors [7]; microfluidics and bioengineering [8,9]; etc. Despite the huge potential of membrane applications, there is a number of factors limiting their use, for example, separation capability (rejection), fouling, and flux decline. Therefore, it is necessary to control the transport and selective properties of a membrane in order to preserve it from any influence caused by these limitations. There are two main ways to affect the membrane’s selective properties: changing the structure of pores (the geometry and physico-chemical properties of the surface) [10,11], including using composite membranes [12]) or external exposure (transmembrane potential, external electric fields [13,14,15], pH of the solution [16], temperature, radiation, etc.).

Nanocomposite membranes have been actively developed in the last decade [17]. The involvement of nanostructures can improve the permeability, selectivity, and anti-fouling properties of a membrane for improved filtration processes. One of the most promising approaches to producing such composite materials is the formation of nanoparticles inside the porous structure of a membrane. As with nanotechnology in general [18], there are two main methods [17] for the formation of nanoparticles inside the membranous pores: “top down”—bulk modification through blending (called mixed-matrix membranes) and “bottom up”—surface modification. In the fabrication of bulk-modified nanocomposite membranes, the nanoparticles are dispersed in a homogeneous polymeric precursor solution before the final formation process [19]. However, this method is difficult to use, for example, in the synthesis of inorganic solid membranes. The surface modification technique is the most convenient method in this case [11]. The surface modification technique deals with deposition of nanoparticles onto a membrane.

Silicate (high silica) porous glasses (PGs) are channel-type nanostructures [20] with thermal, chemical and microbiological stability, in combination with controlled surface structural characteristics [21,22,23]. Special attention is worth paying to the PG application for the separation of liquid mixtures by reverse osmosis. This method has found application in water desalination, sanitary household water cleaning, water regeneration from vital function products in space, radioactive salt concentrations, etc. The use of PG materials appears to be rather efficient in medical applications [24,25] (for instance, PG membranes can be used as hemofilters and in an artificial kidney apparatus). PGs can be filled in with different substances, including metal nanoparticles or nanostructures, and/or exposed to laser radiation to ensure their application in optics, microelectronics, microfluidics, sensorics, solar engineering, ecology, etc. (see [26,27,28,29,30,31,32] and the reviews in them).

One of the main advantages of PG materials is their transparency in the visible range of the optical spectrum [33]. This property can be successfully used in various applications related to laser methods, for example, the method of laser-induced chemical liquid-phase deposition of metals from solution on the surface of different dielectrics (LCLD) [34]. Indeed, in this simple and reliable method, the metal reduction reaction proceeds in the local volume of a solution within the focus of the laser beam, resulting in the formation of catalytically and electrocatalytically active micro- and nanostructured metallic and bimetallic materials with a highly developed surface area. In this regard, the high catalytic activity of the manufactured metal structures can be explained by their highly porous nature. Moreover, in contrast to many other analogs, LCLD makes it possible to synthesize metal nanoparticles continuously from a solution containing a salt of the metal of interest, directly in the reaction mixture, almost without changing its composition. Thus, it is possible to produce different micro- and nanostructures based on copper, nickel, cobalt, iridium, gold, platinum, ruthenium and other metals of nanoparticles of various types (monometallic, bimetallic and metal oxides) by changing physical parameters (laser power, laser pulse duration, laser wavelength, scanning speed, etc.) along with the composition and structure of the solution components [35,36,37,38]. Another merit of LCLD is the opportunity to form metal nanoparticles on the dielectric surfaces, for example, glass or glass-ceramics [39]. In this way, one can fabricate composite membranes with unique properties, combining PG materials and the LCLD technique.

In the present work, gold is chosen as the metal that we deposit into the membrane pores by LCLD. First, gold, like silica, is a biologically and chemically inert material, so there is the prospect of applying our results in medical applications. Second, Au nanoparticles have plasmon resonance in the visible part of the optical spectrum, which opens up possibilities for using the obtained materials in the field of supersensitive sensors [40].

The main goal of this work is to synthesize porous glass composite membranes with Au nanoparticles inside the pores (PG/Au composite membrane) by the laser-induced method and to study their ionic transport properties in the model solutions. These studies will provide better understanding of how the composition of composite membranes affects their properties.

## 2. Materials and Methods

### 2.1. PG Material Synthesis

**Figure 1:**
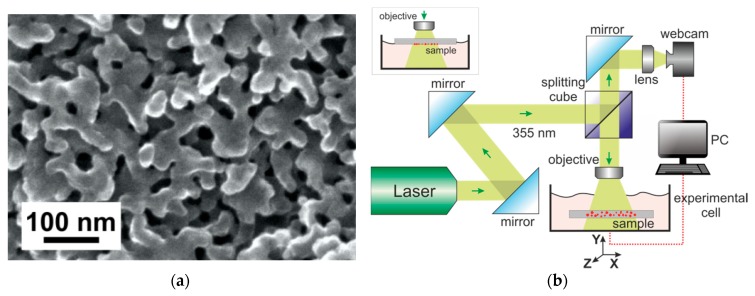
(**a**) Scanning electron microscope image of a porous glass surface; (**b**) Experimental setup for LCLD.

Porous glass (PG) samples (in the form of plane-parallel polished plates 10 × 10 × 1.0 mm^3^ in size) have been prepared by chemical etching of phase-separated sodium borosilicate glass of the following composition (as-analyzed, wt.%): 6.74 Na_2_O, 20.52 B_2_O_3_, 0.15 Al_2_O_3_, 72.59 SiO_2_, with two-frame structure in 3 M HCl and 0.5 M KOH solutions consistently, with subsequent washing in distilled water and drying at 120 °C in an air atmosphere, as described in [41,42]. For studying the porous space parameters, the classical method of equilibrium adsorption and desorption isotherms of nitrogen at 77 K was used. The PG samples’ porosity and average pore diameter were ~0.5 (cm^3^/cm^3^) and 25 nm respectively [43]. Figure 1a shows a typical scanning electron microscopic (SEM) image of a porous glass microstructure, which is a system of tortuosity channels, the material porosity in this case was 52–56%. The porous glasses were manufactured at the Grebenshchikov Institute of Silicate Chemistry of the Russian Academy of Sciences (State Assignment, project no. 0097-2019-0015).

### 2.2. Laser-Induced Liquid Phase Chemical Deposition Process

The experimental scheme shown in Figure 1b was used to form gold nanoparticles by the method of laser-induced chemical liquid-phase deposition of metals from solution (LCLD). The laser beam, reflected in collimating mirrors, enters the beam splitting cube, travels through the focusing system, and is focused on the boundary region between the solution and the sample in the experimental cell, where the photochemical reaction takes place. Then, a portion of the beam reflected from the cell surface is returned to the beam splitter, and subsequently enters the web camera, which controls the focusing process and the reaction in real time. The sample and working solution are placed on the computer-controlled motorized stage. To obtain a homogeneous distribution of the deposition on the sample, a scattering lens with a focal length of 50 mm was used as the objective. The samples were irradiated using a MATRIX pulsed laser (Coherent, Inc. Santa Clara, California, USA) with a diode pump with a wavelength of 355 nm, operating in the single-mode regime, which allows the generation of radiation in a wide power range [44]. The duration of the optical pulses was 25 ns, and the frequency was 2000 Hz. The laser radiation power on the sample was 0.3 watts. For the synthesis of gold nanoparticles, a solution of hydrochloric acid (HAuCl_4_ 3H_2_O, Sigma-Oldrich, St. Louis, Missouri, USA) in water was used as a working solution. Typical concentration values were 1–5 mM.

The diagram (Figure 1b) shows the “solution side” irradiation geometry of the sample. In this case, there are several millimeters of solution above the surface of the sample. However, as our research has shown, this is not the most optimal option for placing the sample. Here, some energy is lost to photochemical reactions in the bulk of the solution. In the case of a heterogeneous photochemical reaction, it is much more efficient to apply the “sample side” lighting geometry (see insert Figure 1b).

### 2.3. Microscopic and Optical Measurement

Scanning electron microscopy (SEM) was used to investigate the morphology of the obtained PG membrane samples. A Merlin (Zeiss, Oberkochen, Gemany) scanning electron microscope with field emission cathode, GEMINI-II electron optics column and oil-free vacuum system was used to get surface images. In addition to the In-lens SE and SE2 secondary electron detectors, the microscope is equipped with a four-quadrant back-scattered electron (AsB) detector and an energy-filtered back-scattered electron (EsB) detector. To determine the elemental composition of the obtained samples, the INCAx-act X-ray microanalysis system (Oxford Instruments, Abingdon, United Kingdom) was used. This method makes it possible to qualitatively determine the composition of the investigated surface from the microscopic region.

The optical properties of the obtained samples were studied on a Lambda 1050 instrument (Perkin Elmer, Waltham, Massachusetts, USA). This spectrophotometer is a dual-beam scanning spectrophotometer with a double monochromator. It allows precision measurements in the 175–3300-nm wavelength range of the following optical characteristics: optical density; reflection and transmission coefficients of liquid and solid substances and materials, including light-scattering inorganic, organic and biological objects.

### 2.4. Ionic Selectivity Measurement

**Figure 2:**
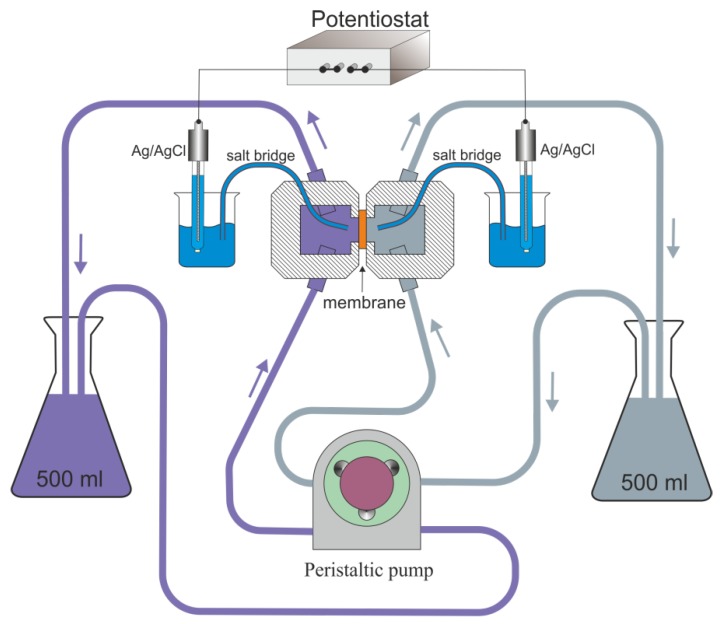
The scheme of potentiometric experimental setup.

The ion perms electivity of the prepared membranes was studied by measuring the potential difference between two electrolyte solutions with different concentrations separated by a membrane at zero current. The scheme of the experimental setup is shown in Figure 2. The laboratory-made electrochemical cell consists of two compartments, between which the membrane is clamped with the help of connection rods and nuts. The cell is made of optically transparent epoxy-based plastic. The sides of compartments facing the membrane are equipped with rubber gaskets. In each of the half-cells, an agar salt bridge connected to the reference 4.2 M Ag/AgCl electrode is located. Salt bridges are necessary for preventing changes of salt concentration in the working solutions. Electrodes are connected to the input of a potentsiostat, which is used as a millivoltmeter in the present configuration (see Figure 2). The potentiostat P-20X (Electrochemical Instruments Ltd., Chernogolovka, Russia) measures the electromotive force (EMF) of the cell in “broken circuit potential measurement” mode. The input impedance of this device is 10^11^ Ω. To prevent the electrical noise, the cell and electrodes are shielded in a metallic box. The system has a common ground closed on the box. In order to eliminate the effect of concentration polarization, in both half-cells, the working solution was pumped using a BT300 (LeadFluid, Baoding, China) peristatic pump with two-channel head (DT15-24, LeadFluid). The flow rate was 10–30 mL/min. The total volume of pumped solution for each of the half cells is 500 mL. The volume of each half-cells was 8 cm^3^.

The measurements were performed in KCl aqueous solution. First, the solution with fixed concentration was placed in both half-cells. The system was kept at a room temperature of 25 °C during 1 h. The measurements were performed by increasing the concentration of KCl in one of the half-cells by consecutive addition of the KCl concentrate (1 M or 3.5 M). The cell EMF was measured continuously throughout the experiment. After each series of experiments, the membrane was placed in deionized water for 24 h to remove the rest of the electrolyte solution from the pores. Measurements for a number of membrane samples produced under the same synthesis conditions demonstrated good reproducibility.

### 2.5. Mathematical Model

The theoretical description of experimental results for ion transport through the membranes is performed with the help of the uniform potential model, which is based on the Navier–Stokes, Nernst–Planck, and Poisson equations [45,46]. The main assumptions of the model, problem statement, and calculation procedure are described below.

Let us consider a membrane, which separates two reservoirs with different concentrations $C_{L}$ and $C_{R}$ of symmetric and monovalent (1:1) aqueous electrolyte ($C_{L}\geq C_{R}$). The electrolyte diffuses from the reservoir with a higher concentration to that with a lower concentration. The reservoirs are maintained at equal constant pressures $\left(P_{L}=P_{R}\right)$, and there is no electric current between them. It is assumed that the membrane surface is charged, and the surface charge density is denoted by σ.

The potential difference between the reservoirs, which develops due to selective ion transport through the pore at zero current, is denoted by $\Delta \Phi =\Phi_{R}-\Phi_{L}$. The magnitude and sign of ΔΦ characterizes the ionic selectivity of the membrane. The case ΔΦ>0 corresponds to the cation-selective membrane, while ΔΦ<0 is related to the anion-selective membrane (strictly speaking, this is valid when the ions have the same diffusion coefficients; otherwise, the diffusion potential can contribute the total membrane potential) [11,47]. The value of membrane potential for the case of an ideally selective membrane is given by the formula [48]:

(1) $$\Delta \Phi_{I}^{\pm }=\pm \frac{R_{g}T}{F}\ln\frac{C_{L}}{C_{R}},$$

where $R_{g}$ is the universal gas constant, T is the temperature, and F is the Faraday constant. The “+” sign corresponds to the cation-selective membrane, and the “–” sign corresponds to the anion-selective membrane.

The membrane is considered as an array of unidirectional cylindrical pores with radius R and length L. Within this approach, it is sufficient to construct a mathematical model of ion transport in a single pore. When such a model is applied to a porous membrane, the average radius calculated from the pore size distribution can be taken as the pore radius in the model. Further, it is assumed that the electric potential Φ, ion concentration $C_{\pm }$, and pressure P are uniform in each cross-section of the pore, so they are functions of coordinate zdirected along the pore. This assumption is valid when the pore radius is comparable or less than the Debye length determined by

(2) $$\lambda =\sqrt{\frac{\varepsilon \varepsilon_{0}R_{g}T}{2C^{\prime }F^{2}}},$$

where $C_{R}\leq C^{\prime }\leq C_{L}$ is the electrolyte concentration, $\varepsilon_{0}$ is the dielectric constant, and ε is relative dielectric permittivity.

Let us define the solvent volume flux (velocity) U, the ion fluxes $J_{\pm }$, the total ion flux $J=J_{+}+J_{-}$, and the ionic charge flux $I=J_{+}-J_{-}$. The dimensionless variables are introduced as follows

(3) $$\begin{array}{c} Z=Lz,\;\Phi =\frac{R_{g}T}{F}\phi ,\;C_{\pm }=C_{0}\;c_{\pm },\;P=C_{0}R_{g}T\;p,\\ U=\frac{D_{+}}{L}u,\;J=\frac{D_{+}C_{0}}{L}j,\;I=\frac{D_{+}C_{0}}{L}i. \end{array}$$

Here, $D_{+}$ and $D_{-}$ are the diffusion coefficients of the cation and anion, respectively, and $C_{0}$ is the characteristic concentration value (hereafter, it is assumed to be 1 mol/m^3^). The dimensionless volume charge density equivalent to the surface charge density σ is defined by

(4) $$X=\frac{2\sigma }{C_{0}FR}.$$

The latter is equal in magnitude and opposite in sign to the ionic volume charge density $c=c_{+}-c_{-}$ due to the electroneutrality condition

(5) $$X=c_{-}-c_{+}.$$

The equations of the homogeneous potential model have the form [45,46]

(6) $$u=\frac{1}{8\alpha }\left[-\frac{dp}{dz}+X\frac{d\phi }{dz}\right],$$

(7) $$j=cu+\frac{1}{2}\left[-(D+1)\frac{dc}{dz}+\left(X(D+1)+c(D-1)\right)\frac{d\phi }{dz}\right],$$

(8) $$i=-Xu+\frac{1}{2}\left[(D-1)\frac{dc}{dz}-\left(c(D+1)+X(D-1)\right)\frac{d\phi }{dz}\right].$$

Here, $c=c_{+}+c_{-}$ is the total concentration of cations and anions; $D=D_{-}/D_{+}$ is the ratio of ion diffusion coefficients, and $\alpha =\mu D_{+}{\left(C_{0}R_{g}TR^{2}\right)}^{-1}$ is the dimensionless parameter determined by the viscosity of the solution. Equations (6)–(8) can be solved with respect to the derivatives dp/dz, dc/dz, dϕ/dz to obtain a system of three differential equations. These equations are derived from full 2D Navier–Stokes, Nernst–Planck, and Poisson equations, which are simplified by taking into account the pore geometry (*R << L*) and assuming that all quantities are uniform in the pore cross-section, see [46] for the details. Equations (6)–(8) relate the volume flux u, total ion flux j, and ionic charge flux i to the pressure, concentration, and potential gradients.

Consider the case of zero ion current through the pore (i=0). Without loss of generality, we assume that the potential in the left reservoir with higher concentration is zero, so the potential in the right reservoir with lower concentration coincides with the membrane potential at zero current ΔΦ (at the same time, Δϕ is the corresponding dimensionless value). Further, the pressure in both reservoirs is considered to be zero. We write the conditions inside the pore at the inlet from the side of the reservoir with a higher concentration (at z=0) [45,46]:

(9) $$p(0)=c(0)-2c_{L},$$

(10) $$c(0)=\sqrt{X^{2}+4c_{L}^{2}},$$

(11) $$\phi (0)=\phi_{0}.$$

Here, $\phi_{0}$ is the Donnan potential jump at the pore inlet. The corresponding concentrations and osmotic pressure jumps are described by conditions (9) and (10). Given that $c_{\pm }(0)=c_{L}\exp(\mp \phi_{0})$, and substituting these equations in (5), we obtain the equation to determine the potential $\phi_{0}$

(12) $$X=2c_{L}\sinh\phi_{0}.$$

We now write the conditions inside the pore at the outlet from the side of the reservoir with a lower concentration (at z=1):

(13) $$p(1)=c(1)-2c_{R},$$

(14) $$c(1)=\sqrt{X^{2}+4c_{R}^{2}},$$

(15) $$\phi (1)=\phi_{1}.$$

The difference between osmotic pressure jumps, just at the pore outlet (z=1) and pore inlet (z=0), results in an osmotic pressure gradient (first term in the right-hand side of Equation (6)), which drives the osmotic flow from the reservoir with lower salt concentration to the reservoir with higher salt concentration. The flow can be also driven by electroosmosis, which results from the impact of the electric field on the uncompensated ionic charge (second term in the right-hand side of Equation (6)).

The relation similar to Equation (12) at z=1 has the form

$$X=2c_{R}\sinh\left(\phi_{1}-\Delta \phi \right).$$

and allows us to determine the membrane potential at zero current:

(16) $$\Delta \phi =\phi_{1}-\mathrm{arcsinh}\left(\frac{X}{2c_{R}}\right).$$

The problem is solved numerically as follows. First, the initial approximations for fluxes u and j are set. Then, the potential $\phi_{0}$ is determined from (12), and boundary conditions (9)–(11) are set at z=0. Then, differential Equations (6)–(8) are integrated numerically using the Runge–Kutta–Merson method of 5th order from z=0 to z=1. The values of fluxes u and j are corrected in order to satisfy boundary conditions (13) and (14) and used for integration of equations at the next step. The iterations are performed until the values of fluxes converge to some constant values with required accuracy. Finally, the membrane potential is calculated from (16).

It should be noted that, when the pore radius is larger than the Debye length (2), the assumption of uniform potential and ion concentrations in the pore cross-section is no longer valid. However, the increase in pore radius decreases the effective volume charge (4), thus the pore size effects can still be described by the presented uniform potential (UP) model. Comparison between values of membrane potential between uniform potential (1D) and space charge (2D) models [11,49] shows good agreement, even when the pore radius exceeds the Debye length.

The described model is valid when the concentrations $C_{L}$ and $C_{R}$ are maintained just at the membrane surface. Experimentally, it is provided by pumping the solutions through half-cells separated by the membrane (see Figure 2). However, at high concentration differences between the cells, the enhanced diffusion flux leads to the formation of concentration boundary layers (BL) near the membrane surface. At the high (low) concentration side, the concentration just near the membrane decreases (increases), so the effective concentration difference is reduced. In this work, we will employ the presented model for describing the experimental data at relatively low concentration differences between half-cells (i.e., when the effect of boundary layers on the membrane potential is weak; see Section 3.2).

The fitting of the measured experimental data to the theoretical model is performed by minimizing the sum of squared errors between the theoretical and experimental points. For a one-parameter fitting, the golden section method is used, while for multi-parameter fitting the Nelder–Mead method is employed.

## 3. Results and Discussion

### 3.1. Glass/Au Composite Membranes Synthesis and Analysis

In the first stage of the study, experiments were carried out to synthesize gold nanoparticles on the smooth surface of the cover glass to better understand the processes of laser synthesis. For this reason, an aqueous solution of chloroauric acid (HAuCl_4_ + H_2_O) was prepared. It is known that chloroauric acid completely dissociates in water into ions H^+^ and [AuCl_4_]^−^. This working solution is quite stable and can be stored in a dark place for a long time. Our studies have shown that the absorption spectrum of this solution in the visible region of the optical spectrum remains unchanged for at least a month. This means that photochemical reactions with the formation of complexes do not take place in solution. Thus, the concentration of ions remains constant. It is also known [50] that the addition of acetone to a solution can lead to a more ordered synthesis of nanoparticles. However, in our case, the addition of even a small amount of acetone (100 µL per 100 mL of solution) led to rapid degradation of the solution. The solution was dark, and precipitate was observed within 5–10 h. All further experiments were carried out only in aqueous solution.

**Figure 3:**
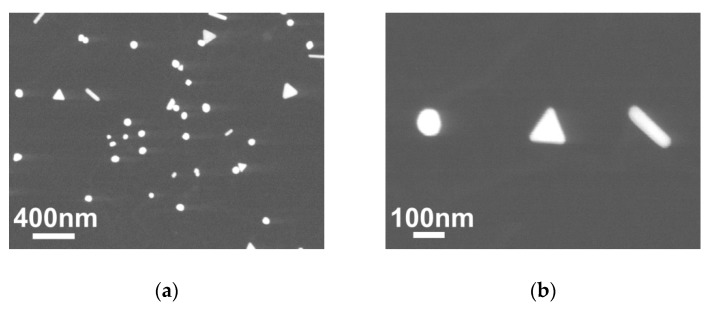

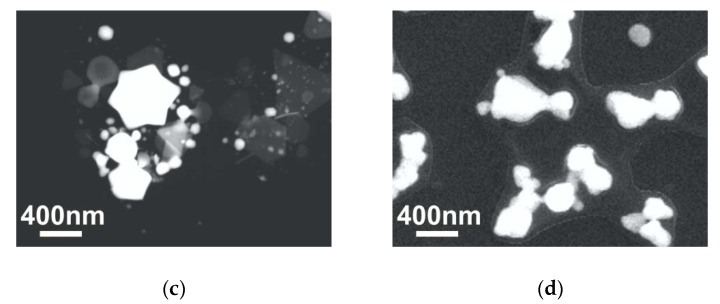
Scanning electron microscope image of a cover glass surface with the LCLD-synthesized Au nanoparticles under different conditions: (**a,b**) exposure time—1 min, solution concentration—1 mM; (**c**) exposure time—5 min, solution concentration—1 mM; (**d**) exposure time—5 min, solution concentration—5 mM.

Typical SEM images of gold nanoparticles obtained by LCLD method on the surface of the cover glass are shown in Figure 3. From the presented images, it can be seen that at low concentrations of the precursor (1 mM) triangular, round and wire single crystal gold nanoparticles are formed. Characteristic particle sizes correspond to 50 nm (in one direction). When the exposure time is increased, the particles are “collected” into a new type—stars (Figure 3c) indicating some level of mobility of the particles on the surface during synthesis. Increasing the concentration of the precursor to 5 mM and the synthesis time leads to the formation of irregular precipitates and loss of crystal structure (Figure 3d).

The mechanism of nanoparticle formation from an aqueous gold tetrachlorourate solution is well studied [51,52,53]. It can be described by several simple equations:

(HAu^3+^Cl_4_)→(HAu^3+^Cl_4_) under *h*ν, (17)

(HAu^2+^Cl_4_)→(HAu^2+^Cl_3_∙∙∙Cl), (18)

(HAu^2+^Cl_3_∙∙∙Cl)→HAu^2+^Cl_3_ + Cl, (19)

2HAu^2+^Cl_3_→HAu^3+^Cl_4_ + HAu^+^Cl_2_, (20)

HAu^+^Cl_2_ →Au^0^ + HCl + Cl under *h*ν, (21)

nAu^0^→(Au^0^)n, (22)

During irradiation by UV light (353 nm), the excited Au^3+^ forms the caged Au^2+^ complex (Equations (17) and (18)). This complex then dissociates (Equation (19)) and the unstable Au^2+^ quickly disproportionates to form Au^+^ and Au^3+^ (Equation (20)). Au^+^ absorbs another photon to form Au^0^ (Equation (21)). Au^0^ associates to form nuclei and the AuNPs (Equation (22)). We assume that, at the final stage of the particle formation process, Au^+^ is adsorbed onto the glass surface and particle formation occurs. It is important to note that the glass surface is charged negatively in an aqueous solution [54]. On the other hand, in contrast to works [55,56] where complex, multicomponent mixtures are used as precursors, we use a simple two-component working solution in the nanoparticle synthesis technique.

**Figure 4:**
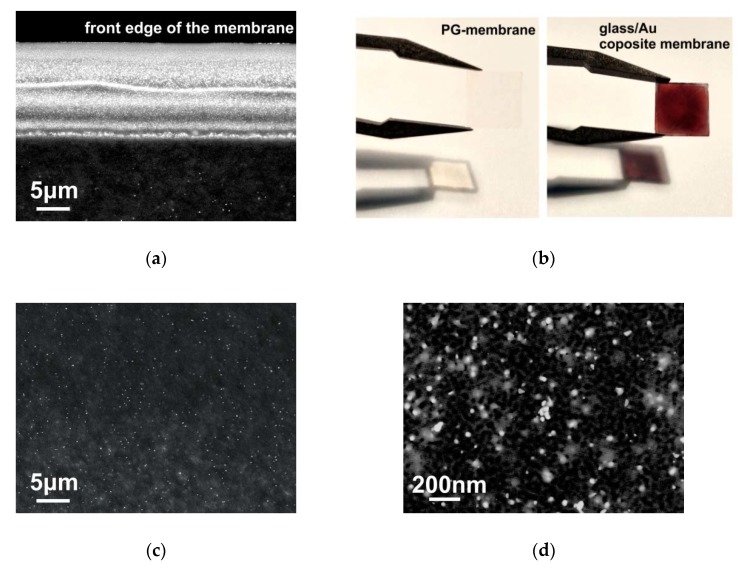
(**a**) SEM image of the cross-section of the Glass/Au composite membrane obtained in “solution-side” irradiation geometry; (**b**) snapshot of the obtained membrane samples; (**c,d**) SEM image of the cross-section of the Glass/Au composite membrane obtained by multiple irradiation technique.

At the next stage, the described nanoparticle synthesis technique was applied to form gold particles inside the pores of PG membranes. Initially, the synthesis was carried out in “solution side” irradiation geometry. It was assumed that particle synthesis would occur uniformly throughout the membrane when the membrane was immersed in in a cell with a working solution. However, the microscopy studies of the membrane cross-section (see Figure 4a) showed that with this configuration of the experimental setup, particles are formed predominantly in the near-surface layers of the membrane. This fact is explained by the intensity of the chemical reaction, and, as a result, the significant absorption of laser radiation in the near-surface layer. In other words, all light flux energy is absorbed at the front edge of the membrane.

In order to obtain samples with a homogeneous distribution of gold particles within the PG - membranes, the following synthesis procedure was proposed. Initially, the PG membrane was impregnated with 1 mM aqueous solution of chloroauric acid (HAuCl_4_ + H_2_O) for 20 min. The impregnation time may vary, but long-term impregnation has been shown to improve the result. Next, the membrane was placed on a glass slide, which was fixed on an object stage. It is important to note that the use of metal devices and tweezers in the process of transporting and manipulating the membrane is impossible, because the metal surface has a high chemical activity in the working solution. The stage was located 4 cm from the objective lens, which scattered the laser radiation reflected from beam splitter (see Figure 1b). The laser spot was pre-tuned on an empty stage, after which a shutter blocking radiation was placed. After positioning the sample, the shutter opened, and the sample was illuminated by laser irradiation for 1 min. Optimal parameters of optical radiation: wavelength 355 nm; pulse time 20 ns; pulse frequency 2000 Hz; radiation power 0.3 watts. After irradiation, the sample was again immersed in the working solution for impregnation for 20 min. The whole procedure was repeated 15 times. It should be emphasized that the sample was turned over with respect to radiation every two stages of synthesis. Thus, the membrane was irradiated on both sides. An important difference between such a synthesis scheme and the one described above is that in this case the sample does not sink to the bottom of the cell filled with the solution. The entire solution is contained within the membrane. There is no radiation-absorbing solution layer above the membrane surface. Thus, the formation of particles in the near-surface layer is not observed. Figure 4a shows typical images of the cross-section of the PG membrane obtained by this procedure. Gold nanoparticles look like bright white dots (see Figure 4c,d). There is a uniform distribution of particles over the membrane volume. The resulting PG/Au composite membrane acquires a purple color (Figure 4b), which is explained by the plasmonic properties of gold nanoparticles.

**Figure 5:**
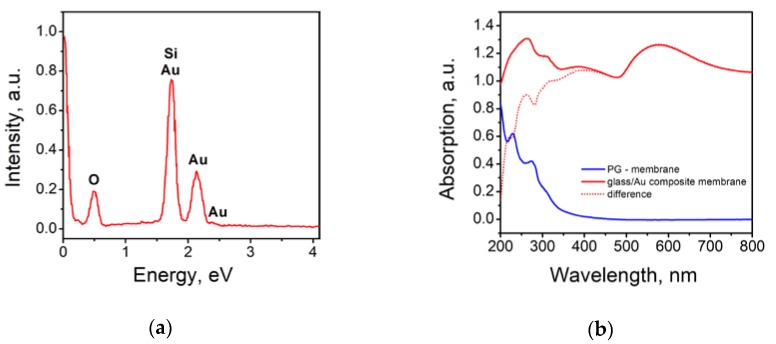
(**a**) Spectrum of characteristic X-ray emission of Glass/Au composite membrane; (**b**) Optical absorption spectrum of a pure PG membrane (blue line), and Glass/Au composite membranes with gold nanoparticles (red line).

To determine the qualitative composition of the synthesized composite, an X-ray surface microanalysis was performed. A typical spectrum of membrane cross-section is shown in Figure 5a. Pronounced peaks corresponding to silicon, oxygen and gold can be observed on the spectrum, which proves the golden nature of the nanoparticles. Additionally, optical absorption spectra of the obtained composites were recorded. The absorption spectra show a broad peak with a maximum at a wavelength of about 580 nm. According to published data [57,58], such absorption is characteristic of particles with an average diameter of 20–30 nm, which confirms the SEM data. It should be noted that the average pore size in the PG membrane is 25 nm, so the average size of gold nanoparticles cannot exceed this value.

### 3.2. Study of Ion Transport Properties of the Glass/Au Composite Membranes

**Figure 6:**
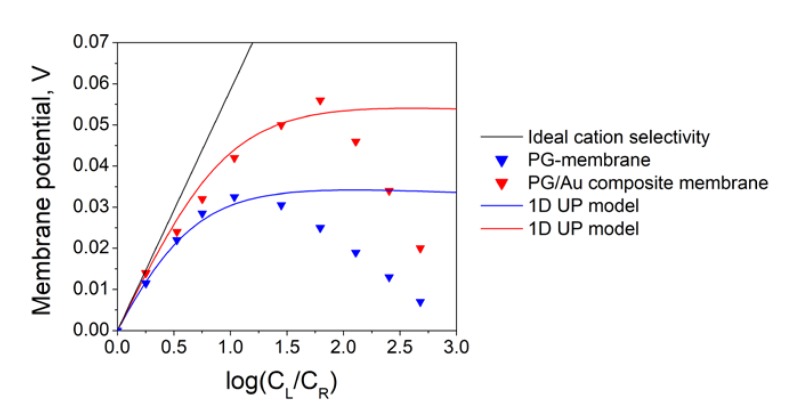
Dependence of the membrane potential on the logarithm of the ratio of solution concentrations on both sides of the synthesized membrane (points—experimental data; lines—the results of mathematical modeling).

We have studied the ionic selectivity of PG membrane and PG/Au composite membranes by measuring the potential difference between two half-cells with different KCl concentrations at zero current (Figure 2). The concentration ($C_{R}$) in one half-cell was fixed at 1 mM, while the concentration in another half-cell ($C_{L}$) was varied from 1 mM up to 3.5 M. Thus, the dependence of the membrane potential on the logarithm of the ratio of solution concentrations on both sides of the membrane was measured (see Figure 6). The black line in Figure 6 is related to (Equation (1)) and describes the case of an ideal cation-selective membrane.

The contact of a PG membrane with an electrolyte leads to the formation of surface charge by adsorption of ions to the membrane surface. As a result, the membrane acquires ionic selectivity provided that the pore radius is comparable with the Debye length (the latter is 9.6 nm for concentration of 1 mM). The PG membranes prepared in this work consist of SiO_2_, the surface charge of which is determined by the solution pH. The latter was 6–7 during the measurements. In this case, the negative charge should accumulate on the surface of silica due to adsorption of anions [54,59]. Therefore, one can expect that PG membranes would be cation-selective.

The results for the PG membrane presented in Figure 6 show that the potential difference between half-cells with low and high electrolyte concentration is positive. It means that the membrane is cation selective. The absolute value of the membrane potential first increases with the increase in concentration ratio, then reaches maximum, and finally decreases. The decrease of membrane potential at high concentration differences can be explained by the strong diffusion, which leads to the formation of concentration boundary layers near the membrane, and, consequently, a decrease in the effective concentration difference. Due to non-linear dependence of PG membrane potential on the logarithm of concentration ratio, the transference numbers for cations and anions strongly depend on concentration. To calculate the membrane surface charge density, the 1D UP model (6)–(16) was used. In all calculations, the following parameter values were used: pore radius 12.5 nm; pore length 500 µm; temperature 298.5 K; solution viscosity 0.89 mPa s. The remaining parameters are reference data. The surface charge determined for the PG membrane was −2.38 mC/m^2^. It should be noted that the 1D UP model describes experimental data only at low concentrations (log*C_L_*/*C_R_* < 1.5) (see the blue line in Figure 6), where the influence of concentration boundary layers is weak. To an adequately describe the entire data set, a modified model with boundary layers should be used. The surface charge values for all membranes are shown in Table 1.

The deposition of Au nanoparticles into the PG membranes pore changes the interaction of membrane surface with the electrolyte solution. It is known that chlorine ions are adsorbed on the surface of gold in aqueous solutions of potassium and sodium chlorides [60]. Thus, due to the formation of gold nanoparticles, the value of the negative charge of the double electric layer inside the pores of the PG/Au composite membrane should increase, and the membrane should become more selective. We observed this result in the experiment (see Figure 6). The maximum value of the membrane potential for the PG/Au composite membrane was near 58 mV, while for the PG membrane it was only 30 mV. For the theoretical description of the experimental data, the 1D UP model was used. As in the case of the PG membrane, a considered model works only in the low concentration range for a PG/Au composite membrane (log*C_L_*/*C_R_* < 2.0). The calculated surface charge in this case was −5.42 mC/m^2^. Thus, we can conclude that the synthesis of gold nanoparticles inside the pores of the PG membrane leads to the increase in membrane surface charge density by about two times, and, consequently, enhances their selectivity.

## 4. Conclusions

In this work, we have proposed a novel type of ion-selective Glass/Au composite membrane based on porous glass, which combines the advantages of porous media and promising selective properties. The latter are achieved by depositing gold nanoparticles into the membrane pores by the laser-induced liquid phase chemical deposition technique. It was shown that it is possible to deposit gold nanoparticles on a glass surface using a simple set of reagents and a simple experimental technique. For the synthesis of PG/Au composite membranes, we used PG materials with porosity of 0.5 (cm^3^/cm^3^) and average pore diameter of 25 nm. Inside the pores, gold nanoparticles with an average diameter of 25 nm were formed, which was confirmed by optical and microscopic studies.

To study the transport and selective properties, the potentiometric method was applied using an electrochemical cell separated by a membrane into two halves. The membrane potentials of the synthesized samples were measured. The maximum value of the membrane potential for the PG/Au composite membrane was near 58 mV, while for the PG membrane it was only 30 mV. The theoretical description of experimental results on ion transport through the membranes was performed with the help of 1D Uniform potential (UP) model, which is based on the Navier–Stokes, Nernst–Planck, and Poisson equations. It was calculated that the formation of gold nanoparticles inside the membrane pores leads to an increase in the surface charge from −2.75 mC/m^2^ to −5.42 mC/m^2^.

The methods proposed in this work allow the creation of a whole family of composite materials based on porous glasses. In this case, conceptually, the synthesis of these materials will differ only in the selection of initial precursors. The potential applications of produced PG/Au composite membranes include nano- and ultrafiltration as well as separation of charged species.

## Figures and Tables

**Table 1:** Surface charge and diffusion boundary layer thickness calculated by different models for the studied membranes.

Membrane Type	Model	Surface Charge
PG membrane	1D UP model	−2.36 mC/m^2^
PG/Au composite membrane	1D UP model	−5.42 mC/m^2^

## Notation List

Dimensionless quantities are given brackets along with the corresponding dimensional ones.

Φ (ϕ)	electrical potential, V
C (c)	ion concentration, mol/m^3^
P (p)	pressure, Pa
Z (z)	space coordinate, m
U (u)	volume flux (velocity), m/s
J (j)	ion flux, mol/m^2^ s
I (i)	charge flux, mol/m^2^ s
ΔΦ (Δϕ)	potential difference, V
F	Faraday constant, C/mol
T	temperature, K
$R_{g}$	universal gas constant, J/kg K
ε	relative dielectric permittivity
$\varepsilon_{0}$	vacuum permittivity, F/m
λ	Debye length, m
σ	surface charge, C/m^2^
X	volume charge, mol/m^3^
$D_{\pm }$	ion diffusion coefficients, m^2^/s
D	ratio of ion diffusion coefficients $D=D_{-}/D_{+}$
$C_{0}$	characteristic concentration, 1 mol/m^3^
R	pore radius, m
L	pore length, m
α	dimensionless parameter
Subscripts	
+	cation
–	anion
L	left reservoir
R	right reservoir
I	ideal
0	pore inlet
1	pore outlet
Superscripts	
+	cation selectivity
–	anion selectivity
